# Chromosomal engineering of inducible isopropanol- butanol-ethanol production in *Clostridium acetobutylicum*

**DOI:** 10.3389/fbioe.2023.1218099

**Published:** 2023-06-16

**Authors:** Bunmi B. Omorotionmwan, Hengzheng Wang, Jonathan P. Baker, Krzysztof Gizynski, Minyeong Yoo, Cynthia Akaluka, Ying Zhang, Nigel P. Minton

**Affiliations:** ^1^ Clostridia Research Group, BBSRC/EPSRC Synthetic Biology Research Centre (SBRC), Biodiscovery Institute, School of Life Sciences, The University of Nottingham, Nottingham, United Kingdom; ^2^ NIHR Nottingham Biomedical Research Centre, Nottingham University Hospitals NHS Trust and the University of Nottingham, Nottingham, United Kingdom

**Keywords:** auxotroph, orthogonal, inducible system, isopropanol-butanol-ethanol, *Clostridium*, allele-coupled exchange

## Abstract

The use of environmentally damaging petrochemical feedstocks can be displaced by fermentation processes based on engineered microbial chassis that recycle biomass-derived carbon into chemicals and fuels. The stable retention of introduced genes, designed to extend product range and/or increase productivity, is essential. Accordingly, we have created multiply marked auxotrophic strains of *Clostridium acetobutylicum* that provide distinct loci (*pyrE*, *argH*, *purD*, *pheA*) at which heterologous genes can be rapidly integrated using allele-coupled exchange (ACE). For each locus, ACE-mediated insertion is conveniently selected on the basis of the restoration of prototrophy on minimal media. The *Clostridioides difficile* gene (*tcdR*) encoding an orthogonal sigma factor (TcdR) was integrated at the *pyrE* locus under the control of the lactose-inducible, *bgaR::*P_*bgaL*_ promoter to allow the simultaneous control of genes/operons inserted at other disparate loci (*purD* and *pheA*) that had been placed under the control of the P_*tcdB*_ promoter. In control experiments, dose-dependent expression of a *catP* reporter gene was observed with increasing lactose concentration. At the highest doses tested (10 mM) the level of expression was over 10-fold higher than if *catP* was placed directly under the control of *bgaR::*P_*bgaL*_ and over 2-fold greater than achieved using the strong P_*fdx*_ promoter of the *Clostridium sporogenes* ferredoxin gene. The utility of the system was demonstrated in the production of isopropanol by the *C. acetobutylicum* strain carrying an integrated copy of *tcdR* following the insertion of a synthetic acetone operon (*ctfA/B, adc*) at the *purD* locus and a gene (*sadh*) encoding a secondary dehydrogenase at *pheA*. Lactose induction (10 mM) resulted in the production of 4.4 g/L isopropanol and 19.8 g/L Isopropanol-Butanol-Ethanol mixture.

## 1 Introduction

The production of chemicals and fuels through the recycling of biomass-derived carbon using biological fermentation processes has an increasingly important role to play in displacing environmentally damaging petrochemical feedstocks. Although some microorganisms naturally produce the requisite chemicals, metabolic engineering has a major role to play, both in enhancing productivity and extending product streams. Largely reliant on the provision and optimized expression of the necessary encoding genes, engineered strains invariably carry the requisite pathways on autonomous plasmids. One consequence is that the introduced plasmid, together with the engineered pathway, can be lost due to segregational instability. Although this can be avoided through the provision of a vector encoded antibiotic resistance gene and inclusion in the media of antibiotics, such practices are unwise if the spread of antimicrobial resistance is to be controlled. One solution is to stably integrate the requisite pathways into the genome.

We have previously described a procedure whereby DNA of any size or complexity can be rapidly integrated into the genome, termed Allele-Coupled Exchange (ACE). Initially exemplified in *Clostridium acetobutylicum* (Heap et al., 2012), its deployment has been largely confined to anaerobic bacteria and in particular clostridia. Although more generally associated with human and animal disease, clostridia are of increasing industrial importance, and in particular solvent producing *Clostridium* species such as *Clostridium acetobutylicum* and *Clostridium beijerinckii,* have been target chassis for strain engineering towards improved solvent production (Mermelstein and Papoutsakis, 1993; Jang et al., 2012; Jang et al., 2013; Ehsaan et al., 2016; Nguyen et al., 2018; Zhang et al., 2018; Xin et al., 2020).

The preferred embodiment of the ACE method developed in *C. acetobutylicum* targets the native *pyrE* gene which encodes orotate phosphoribosyltransferase and is part of the pyrimidine biosynthesis pathway. ACE is initially employed to replace the wildtype gene with a mutant allele lacking codons from the 3′ end of the structural gene. The mutant created becomes an uracil auxotroph resistant to 5-fluoro-orotate (FOA) and can be rapidly converted back to wildtype using a purpose built ACE correction vector, selecting for uracil prototrophy. Concomitant with *pyrE* repair, additional DNA fragments can be delivered to the genome. In the current report we have extended the number of auxotrophic mutant loci at which ACE may be employed to introduce cargo to three additional alleles, namely, deletion mutants of *purD, argH* and *pheA.* The former encodes phosphoribosylamine–glycine ligase, involved in the *de novo* synthesis of purine nucleotides. The *argH* and *pheA* genes code for argininosuccinate lyase and prephenate dehydratase which are involved in arginine and phenylalanine biosynthesis, respectively. The equivalent deletions to that of *pyrE* in these genes were made using a newly developed knock-out vector that allows the positive selection of in-frame deletions. The resultant *∆purD*, *∆argH* and *∆pheA* mutants respectively required supplementation with a purine cocktail (comprising adenine, guanine and hypoxanthine), arginine or phenylalanine to support growth on minimal media. The successful ACE-mediated integration of DNA at these sites using the requisite correction vectors is simply selected by the conversion of integrants to prototrophy.

The provision of three different loci in the same cell allows the stable, independent integration of different components of a metabolic pathway. Here we use the one locus (*pyrE*) to integrate an orthogonal sigma factor responsible for the expression of the genes inserted at two other loci (*purD* and *pheA*) which together bring about the production of acetone and isopropanol. This arrangement allows all of the genes of a pathway to be under the same regulatory control despite their disparate location around the genome.

## 2 Materials and methods

### 2.1 Bacteria strains and plasmids

All bacteria strains and plasmids used are given in Table 1. Plasmids may be sourced from www.plasmidvectors.com.

**TABLE 1:** Bacterial strains and plasmids used.

Bacteria/Plasmids	Description/Features	Source
Strains^a^		
*E. coli* Top10	Cloning and plasmid storage host	Invitrogen, Ltd
*E. coli* Top10 (pAN2)	Top10 carrying pAN2 methylation plasmid	Heap et al. (2007)
*C. acetobutylicum* ATCC 824	WT parental strain	Rostock, Germany
*C. acetobutylicum* Δ*pyrE::dif*	*ΔpyrE* mutant created by ACE	Ehsaan et al. (2016)
*C. acetobutylicum* Δ*pheA::dif*	*ΔpheA* auxotrophic mutant	This study
*C. acetobutylicum* Δ*purD::dif*	*ΔpurD* auxotrophic mutant	This study
*C. acetobutylicum* Δ*argH::dif*	*ΔargH* auxotrophic mutant	This study
*C.acetobutylicum ΔpyrE::dif, ΔargH::dif*	*ΔpyrE* & *ΔargH* double auxotroph	This study
*C. acetobutylicum ΔΔpyrE::dif, ΔpurD::dif*	*ΔpyrE* & *ΔpurD* double auxotroph	This study
*C. acetobutylicum ΔpyrE::dif, ΔpurD::dif, ΔpheA::dif*	*ΔpyrE, ΔpurD, ΔpheA* triple auxotroph	This study
*C. acetobutylicum* 824BO1	*bgaR*-P_*bgal*_-*tcdR* integrated at the *pyrE* locus	This study
*C. acetobutylicum* 824BO2	*bgaR*-P_*bgal*_-*tcdR* at *pyrE* locus, P_*tcdB*_ -*ctfA/B-adc* at the *purD* locus, WT *pheA* locus	This study
*C. acetobutylicum* 824BO3	*bgaR*-P_*bgal*_-*tcdR* at *pyrE* locus, P_*tcdB*_-*ctfA/B-adc* at the *purD* locus and P_*tcdB*_ -s*adh* at *pheA*	This study
**Plasmid**		
pMTL82254	Modular reporter vector for clostridia; pBP1 Gram-positive replicon, ColE1+*tra* Gram-negative replicon, *ermB* antibiotic marker with *catP* reporter	Heap et al. (2009)
pMTL85121	Modular vector for clostridia; pIM13 Gram-positive replicon, p15a + tra Gram-negative replicon, *catP* antibiotic marker and multiple cloning site	Heap et al. (2009)
pMTL_SC7515	Knock-out plasmid for clostridia; pIM13 Gram-positive replicon, *codA* in backbone; ColE1 Gram-negative replicon, *catP* antibiotic marker and multiple cloning site	Cartman et al. (2012)
pMTL-KG146	Novel Suicide Knock-out plasmid for clostridia; *codA* in backbone; ColE1 replicon, marker cassette comprising *catP* and *pyrE*flanked by identical repeats of random 29 bp sequence	This Study
pMTL-KG147	Novel Suicide Knock-out plasmid for clostridia; *codA* in backbone; ColE1 replicon, marker cassette comprising *catP* and *pyrE* flanked by identical repeats of the predicted *C. acetobutylicum dif* sequence.	This Study
pMTL-KG147_argH	pMTL-KG147 with homology arms to mediate *argH* knock-out	This Study
pMTL-KG147_pheA	pMTL-KG147 with homology arms to mediate *pheA* knock-out	This Study
pMTL-KG147_purD	pMTL-KG147 with homology arms to mediate *purD* knock-out	This Study
pMTLME6C	*pyrE* complementation plasmid and backbone for *purD* and *pheA* complementation/expression plasmids pIM13/ColE1 replicon; Cm^R^/Tm^R^	Ng et al. (2013)
pMTL-BO1C	*purD* complementation plasmid based on pMTLME6C	This Study
pMTL-HZ1C	*pheA* complementation plasmid based on pMTLME6C	This Study
pMTL-HZ2C	*argH* complementation plasmid based on pMTLME6C	This Study
pMTL82254::PtcdB	The reporter plasmid pMTL82254 in which the *catP* gene has been placed under the transcriptional control of the P_*tcdB*_ promoter.	This Study
pMTL82254::Pfdx	The reporter plasmid pMTL82254 in which the *catP* gene has been placed under the transcriptional control of the P_*fdx*_ promoter*.*	This Study
pMTL82254::PbgaL	The reporter plasmid pMTL82254 in which the *catP* gene has been placed under the transcriptional control of the *bgaR*-P_bgal_ inducible promoter system.	This Study
pMTL-HZ13-HZ-tcdR	ACE complementation vector for the *pyrE* locus carrying *tcdR* under the control of the *bgaR*-P_bgal_ inducible promoter system.	This Study
pMTL-BO16-PtcdB-ctfAB-adc	ACE complementation vector for the *purD* locus carrying the genes necessary for acetone production, *ctfAB* and *adc.*	This Study
pMTL-HZ15-PtcdB-SadH	ACE complementation vector for the *pheA* locus carrying the gene necessary for isopropanol production, *sadh*.	This Study

^a^ **—**While auxotrophic mutants with either a *dif* or *ran* scar were made, for consistency all the in-frame deletion mutants used in the study carry the *dif* sequence.

### 2.2 Culture conditions

*C. acetobutylicum* ATCC 824 and its derivatives were grown in the Clostridial Growth Medium (CGM) of Hartmanis and Gatenbeck (1984), P2 minimal medium (Baer et al., 1987) or 2xYeast Tryptone Glucose (YTG) broth (Mermelstein et al., 1992). Growth was at 37°C in an anaerobic cabinet (MG1000 Anaerobic Workstation, Don Whitley Scientific Ltd) containing an atmosphere of 80% nitrogen, 10% hydrogen and 10% carbon dioxide. *Escherichia coli* strains were grown in Luria Bertani (LB) Medium (Sambrook et al., 1989) at 37°C. When necessary, solid and liquid media for *E. coli* was supplemented with 25 μg/mL and 12.5 μg/mL of chloramphenicol, respectively. Tetracycline was always added at a concentration of 10 μg/mL. Clostridial media was supplemented when needed with 15 μg/mL of thiamphenicol and 10 μg/mL of erythromycin. Pyrimidine and purine auxotrophic mutants were respectively supplemented with 20 μg/mL of uracil and a purine cocktail made up of equal proportions of adenine, guanine and hypoxanthine. The counter-selective agent 5-fluoroorotic acid was included in agar media when needed at 400 μg/mL.

### 2.3 Transformation of *C. acetobutylicum*

A heavy loop of *C. acetobutylicum* was inoculated into 10 mL of 2 × YTG media in serial dilutions and grown in an anaerobic cabinet at 37°C overnight. A conical flask containing 310 mL of 2 × YTG medium was inoculated with 10 mL of the overnight culture representing the lowest dilution that still exhibited good growth and incubated as before until an OD600 of 0.2–0.25 was reached. The culture was transferred to six 50 mL falcon tubes and centrifuged for 10 min at 4°C, 5,000 × g. Pelleted cells were resuspended in 40 mL of ice-cold electroporation buffer (EPB) by shaking or gentle pipetting. EPB comprised 270 mM Sucrose, 5 mM Na_2_HPO_4_ and 5 mM NaH_2_PO_4_ and pH was adjusted to 7.4. Cell suspensions were centrifuged as before in six separate centrifuge tubes and the pellets obtained resuspended in 15 mL of ice cold EPB. The resuspended contents of three tubes were combined into a single centrifuge tube and centrifuged as before. The pellets were resuspended in 0.8 mL ice cold EPB by gentle pipetting and then combined. The competent cells were ready to electroporate after adding 10% (w/v) DMSO. The competent cells with 10% DMSO can be aliquoted and stored at −80°C for up to 8 weeks with no change in observable competence. Frozen cells were defrosted in ice for 20 min, then electroporated as described above.

For electroporation, at least 10 μg of methylated plasmid DNA and 590 μL of competent cells were transferred gently into an ice-cold electroporation cuvette (Biorad) and incubated for 2 min on ice. The mixture was electroporated using 2.0 kV, 25 μF and ∞Ω program on a BioRad Gene pulser before addition of 1 mL of warm (37°C) anaerobic 2 × YTG. The culture was transferred into a universal tube containing 9 mL 2 × YTG and incubated for at least 4 h in an anaerobic cabinet at 37°C. Cells were harvested as before at room temperature and pellets were resuspended in 0.5 mL of fresh pre-warmed (37°C) anaerobic 2 × YTG and plated onto CGM agar plates with appropriate antibiotic supplementation.

Prior to transformation, plasmid DNA was purified from *E. coli* TOP10 cells containing plasmid pAN2 (Heap et al., 2007). This plasmid contains the ϕ3TI methyltransferase gene of *B. subtilis* phage ϕ3tI, which protects DNA from *C. acetobutylicum* Cac824I DNA restriction activity (Mermelstein and Papoutsakis., 1993, Heap et al., 2007).

### 2.4 Plasmids and DNA modification techniques

Chromosomal and plasmid DNA preparation, purification of DNA gel fragments and DNA clean-up was undertaken using Qiagen kits, United Kingdom (DNeasy Tissue kit, QIAprep Miniprep kit and QIAquick Gel Extraction kit). Restriction enzymes were from the New England Biolabs and used according to manufacturer’s instructions. Phusion High Fidelity and DreamTaq DNA polymerases (Thermo Fisher Scientific, United Kingdom) were used for PCR amplifications. Agarose gel electrophoresis was carried out as described (Sambrook et al., 1989) and oligonucleotides were synthesized by Merck, UK.

### 2.5 Knock-out plasmid design

**FIGURE 1:**
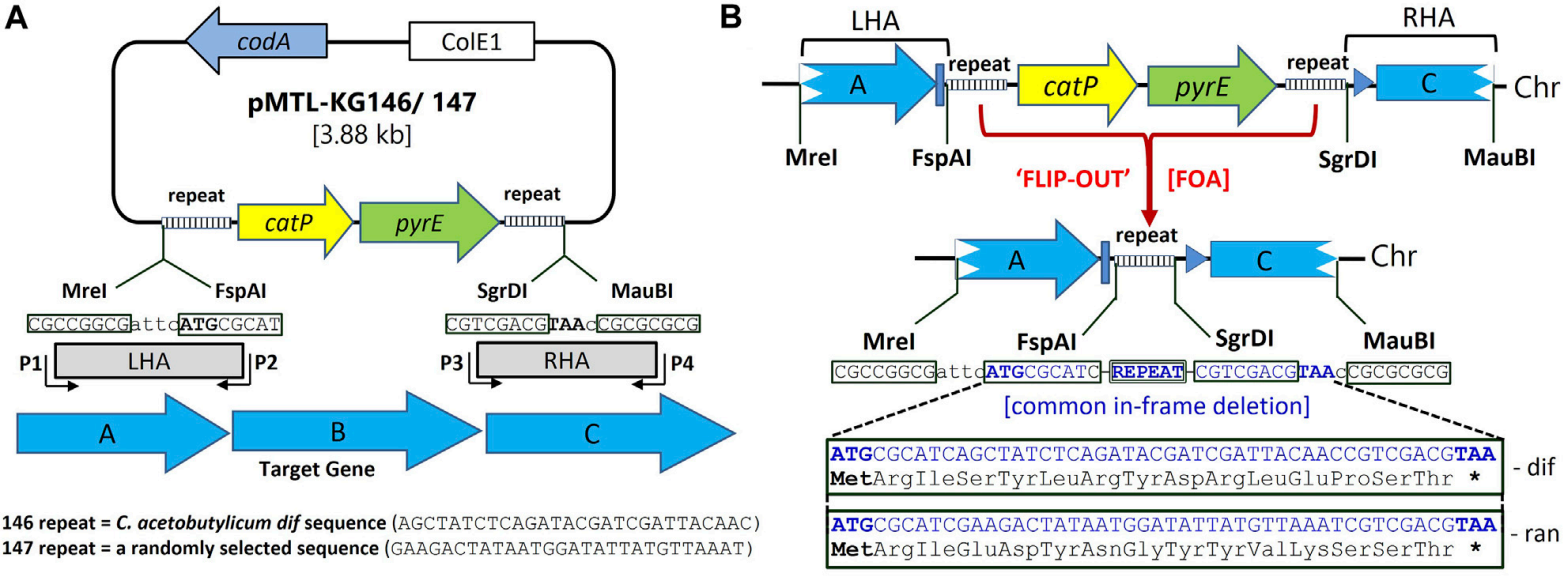
Suicide plasmid, pMTL-KG146/7 for in-frame deletion in clostridia. **(A)** Both plasmids carry the Gram-negative ColE1 replication region but no Gram-positive replicon. They possess the counter-selection markers *codA* (cytosine deaminase) and *pyrE* (orotate phosphoribosyltransferase). The latter is preceded by the positive selection marker *catP* (chloramphenicol acetyl transferase) with the two genes flanked by identical repeat regions. These comprise a random 29 bp sequence (*ran*) in the case of pMTL-KG146, whereas pMTL-KG-147 carries the predicted *C. acetobutylicum dif* sequence. Flanking the repeat sequences are two different pairs of 8 nt recognition sites into which homology arms (HAs) are cloned. These are the sites for the restriction enzymes *Mre*I and *Fsp*AI in the case of the left HA (LHA) and *Sgr*DI and *Mau*BI for the right HA (RHA). The DNA, typically between 500 and 1,000 bp, comprising the LHA and RHA are PCR amplified using region-specific primers P1-P4. The P2 primer is designed such that the ATG of the *Fsp*AI site equates to the start codon of the gene to be deleted (e.g., gene “B” in the example) whereas primer P3 includes the indicated TAA stop codon that follows the *Sgr*DI recognition site. **(B)** Following isolation of the double crossover mutant on media supplemented with thiamphenicol and 5FC, colonies are plated on media containing FOA which selects for those cells that have lost the DNA region encoding *pyrE::catP* as a result of recombination between the two repeat regions. Flip-out of this DNA leads to the presence of a single copy of the repeat region in the genome and the creation of an in-frame deletion in gene “B” encoding a common 15 amino acid peptide. This differs depending on which plasmid is used, pMTL-KG146 or pMTL-KG147.

The knock-out vectors used to generate the required deletion mutants, pMTL-KG146 and pMTL-KG147 (Figure 1A) make use of the two counter-selection markers *codA* (encodes cytosine deaminase) and *pyrE* (encodes orotate phosphoribosyltransferase), as well as a positive selection marker, *catP* (coding for chloramphenicol acetyl transferase and conferring resistance to thiamphenicol). Acquisition of a functional *codA* gene by the clostridial target makes cells sensitive to 5-Fluorocytosine (5FC), whereas the presence of a functional *pyrE* gene renders cells sensitive (^S^) to 5-Fluoroorotic acid (FOA). It follows that cells acquire resistance (^R^) to 5FC and FOA following the respective removal/inactivation of *codA* or *pyrE*. While *codA* resides on the vector backbone, *catP* and *pyrE* are tandemly located within the mutant allele, between the two homology arms (HAs). Moreover, the DNA fragment carrying *catP::pyrE* are flanked by identical, small repeat sequences able to mediate deletion of the entire intervening region (Figure 1A). These equate to the predicted (Carnoy and Roten, 2009) 28 bp *C. acetobutylicum dif* sequence (5′-GAA​GAC​TAT​AAT​GGA​TAT​TAT​GTT​AAA​T-3′) in plasmid pMTL-KG147 and a random (*ran*) 29 bp sequence (5′-AGC​TAT​CTC​AGA​TAC​GAT​CGA​TTA-CAA​CC-3′) in plasmid pMTL-KG146. The *dif* sequence has previously been shown to be highly effective at bringing about Xer recombinase-mediated excision of marker genes from bacterial chromosomes (Bloor and Cranenburgh, 2006). Exploitation of the *pyrE* gene necessitates that the host used for mutant generation has a deletion in its chromosomal copy, equivalent to that previously described (Ehsaan et al., 2016), and is therefore FOA^R^.

The vector design, and in particular the relative positioning of the left and right HAs (LHA and RHA), is such that the deletion event mediated by either repeat results in the creation of a small ORF (Figure 1B). The translational start and stop codon of the encoded peptide are synonymous to those of the gene being targeted for deletion. This is ensured by the PCR amplification of the region of DNA encompassing the LHA using primers that create a distal and proximal *Mre*I (CGCCGGCG) and *Fsp*AI (ATGCGCAT) recognition sites in which the ATG of the latter corresponds to the translational start codon of the gene targeted for deletion. In the case of the RHA, through the use of appropriate oligonucleotide primers, the amplified DNA fragment is designed to encompass flanking recognition sites for *Sgr*DI and *Mau*BI (GTCGACG) such that the recognition site of the latter is immediately followed by a TAA codon that corresponds to the translational stop codon of the gene targeted for deletion. As a consequence of this HA design and use of the created restriction sites for cloning, the subsequent repeat region deletion event leads to creation of a mutant allele comprising an in-frame deletion that encodes a 15 amino acid peptide. Dependent on the repeat region, the encoded peptide is always the same, equating to MRISYLRYDRLQPST in the case of *ran* and MRIEDYNGYYVKSST in the case of the *dif* sequence (Figure 1B).

The two vectors were generated using Gibson assembly. Firstly, primers codA_F, codA_R, colE1_F and colE1_R were used to amplify *catP, codA* and ColE1 from plasmid pMTL_SC7515 (Cartman et al., 2012) with Q5 polymerase; primers catP_F1 (*dif*), catP_F2 (*ran*), catP_R, pyrE_F, pyrE_R1 (*dif*) and pyrE_R2 (ran) were used to amplify *catP* and *pyrE* from pMTL_ME6 (Ehsaan et al., 2016). All PCR fragments were assembled in a four-fragment Gibson assembly. This resulted in pMTL-KG146 and pMTL-KG147 which were transformed in *E. coli* DH5α using heat shock according to New England Biolabs (NEB) instructions. After growth of colonies, PCR screening and miniprep, the correct plasmids were used for the construction of knock-out vectors as described in Supplementary Material.

### 2.6 Target sites for gene integration using ACE

To select alternative genomic loci at which heterologous genes could be integrated using ACE, three essential criteria were required: i) like *pyrE*, they should represent the last gene of an operon, thereby preventing polar effects on downstream genes following subsequent insertion of cargo DNA; ii) their inactivation should result in an easily detectable phenotype, most obviously auxotrophy, and; iii) their restoration should be selectable, most simply through acquisition of prototrophy. Three promising candidates were identified as *pheA* (CAC0217, prephenate dehydratase), *purD* (CAC1396, phosphoribosylamine–glycine ligase) *argH* (CAC0974, argininosuccinate lyase). Their respective inactivation should lead to phenylalanine (Nelms et al., 1992), purine (Truong et al., 2015) and arginine auxotrophy (Reimers et al., 2004).

To act as loci for ACE-mediated cargo insertions, the mutant allele targeted should comprise a gene with a truncated 3‘-end, but which still retains its 5′-end and non-coding region, as exemplified with the *pyrE* made in *C. acetobutylicum* (Heap et al., 2012). Accordingly, the requisite 1,000 bp homology arms were PCR amplified from each gene using primers that incorporated either the restriction recognition sites *Mre*I and *Fsp*AI (Left Homology Arm, LHA) or *Sgr*DI and *Mau*BI (Right Homology Arm, RHA). These sites allowed the subsequent restriction cloning of the HAs into either plasmid pMTL-KG146 or pMTL-KG147 following their appropriate sequential cleavage with *Mre*I/*Fsp*AI and *Sgr*DI and *Mau*BI. For full details of the plasmids constructed, see Supplementary Material. Mutant alleles of the three targeted genes were respectively truncated from codon positions 176, 282 and 302 for genes *pheA, purD* and *argH*.

Mutation of the three genes was undertaken in the *pyrE* mutant. Following its transformation with the three different KO derivatives of pMTL-KG146 and pMTL-KG147, transformants were selected on CBM agar supplemented with either: i) thiamphenicol, or with; ii) thiamphenicol and 5FC. In addition to these selective agents, the media was additionally supplemented with uracil (to compensate for the *pyrE* deletion) and either arginine (for pMTL-KG146_argH and pMTL-KG147_argH), phenylalanine (for pMTL-KG146_pheA and pMTL-KG147_pheA) or a cocktail of adenine, guanine and hypoxanthine (for pMTL-KG146_purD and pMTL-KG147_purD). Colonies that arose on i) represented predominately single crossover mutants, whereas those that arose on ii) were double crossover mutants. In the event no colonies were obtained on ii) then Tm^R^ colonies from i) could be passaged on the media of ii) to obtain the desired double crossover mutant. Following their isolation, the Tm^R^-5FC^R^ mutants were plated onto Tm- and uracil-deficient media containing FOA and, dependent on the gene being targeted, supplemented with arginine, phenylalanine or the purine cocktail. Cells in which the *catP*::*pyrE* cassette flips out, due to recombination between the direct repeats that flank the cassette become resistant to FOA, as now the only *pyrE* allele in the cell is the original mutant one. Their identity as the desired mutant was confirmed by PCR using appropriate gene flanking primers (see Supplementary Table S1) followed by Sanger sequencing of gene fragments. Further confirmation that the *catP::pyrE* fragment had excised was obtained by showing the cells no longer grew on agar media supplemented with Tm.

### 2.7 ACE complementation vectors

It has previously proven possible to deliver cargo DNA into the *C. acetobutylicum* chromosome at the mutant *pyrE* locus concomitant with its restoration to wildtype using the complementation ACE vector pMTL-ME6C (Ehsaan et al., 2016). This plasmid carries a 300 bp Short Homology Arm (SHA), comprising a 5′ truncated *pyrE* allele (missing 13 codons), and a 1,200 bp Long Homology Arm (LHA) encompassing the region of DNA immediately downstream of the *pyrE* gene. Positioned between the two HAs are a number of restriction enzyme recognition sites into which cargo DNA may be cloned. When transformed into an appropriate *pyrE* mutant, the plasmid preferentially integrates through recombination at the larger RHA, to produce faster growing Tm^R^ cells. When these are streaked onto CBM media lacking uracil, it selects cells in which recombination at the LHA has led to restoration of the *pyrE* locus to wildtype (prototrophy) and excision of the plasmid. Concomitant with restoration of *pyrE* any cargo DNA cloned between the restriction sites becomes integrated into the genome immediately downstream of *pyrE.* The use of this system provides an extremely rapid, simple and failsafesimple means of delivering cargo DNA into the genome—restoration of prototrophy cannot arise by means other than the intended integration event. Integration is, however, limited to this one locus.

To extend the sites to which DNA may be delivered, equivalent complementation vectors to pMTL-ME6C were constructed for the three loci, *pheA, argH* and *purD*. These were made by replacing the *pyrE* locus-derived SHA and LHA of pMTL-ME6C. These are respectively flanked by either a *Sbf*I and *Not*I restriction recognition site, or by *Nde*I and *Asc*I sites. Accordingly, the equivalent regions of the *pheA, argH* and *purD* loci were PCR amplified using primers that incorporated the necessary restriction endonuclease recognition sites, and the DNA fragment generated following their cleavage with either *Sbf*I and *Not*I (LHA) or *Nde*I and *Asc*I (RHA) cloned in place of the *pyrE* regions of pMTL-ME6C. In the case of *pheA,* for example, primers pheA_CLHA_F and pheA_CLHA_R were used to amplify the LHA and pheA_CLHA_F and pheA_CLHA_R to amplify the RHA. Full details of the construction of these plasmids is given in the Supplementary Material. The plasmids generated (Supplementary Figure S1) were designated pMTL-HZ1C (*pheA*), pMTL-HZ2C (*argH*) and pMTL-BO1C (*purD*).

### 2.8 Fermentation and metabolite profile analysis

pH-controlled fermentation was carried out in Multifors bioreactors (Infors UK Ltd., UK) with a working volume of 400 mL. CGM medium supplemented with glucose (80 g/L) was sparged with nitrogen for 1 h before inoculation (Dusseaux et al., 2013). Cultures were stirred at 200 rpm, the temperature was set to 35°C, and the pH was maintained above 5.0 by addition of 3 M ammonia solution. The precultures were grown in CGM supplemented with glucose (60 g/L) under strict anaerobic condition and inoculation of pH-controlled fermentations to an initial OD600 ca. 0.2. Lactose induction was performed after 6 h sampling at a final concentration of 10 mM.

Metabolites were determined by HPLC analysis (Dionex UltiMate 3000 HPLC system, ThermoFisher Scientific). Biorad Aminex HPX-87H column (300 mm × 7.8 mm) was used for separation and a refractive index detector (to analyse glucose, ethanol, and butanol) and UV detectors (absorbance at 210 nm for acids, 280 nm for acetone) were used for detection, respectively. Samples were diluted two times and run at a flow rate of 0.5 mL/min at 20°C in 5 mM H_2_SO_4_ mobile phase for 1 h.

### 2.9 CAT enzyme assay

Strains were grown in 2xYTG media supplemented with erythromycin, samples were collected at various time points and centrifuged at 13,000 × g for 10 min at 4°C and the pellets stored at −80°C. Lactose induction was performed at mid-log phase. Cell pellets were re-suspended in 0.5 mL of PBS supplemented with Roche Complete protease inhibitor cocktail tablets and lysed by sonication using a Sonifier S-450D (Branson Ultrasonics Corp., Danbury, CT, United States). The lysates were centrifuged at 13,000 × g for 30 min at 4°C and transferred into new Eppendorf tubes for further analysis. CAT enzyme assay was undertaken according to the method described by Shaw (1975). A quartz cuvette was prepared containing 540 µL of 100 mM Tris buffer (pH 7.8), 200 µL of 2.5 mM DTNB Solution (5,5′-Dithio-bis (2-Nitrobenzoic Acid) in 100 mM Tris buffer, pH 7.8), 200 µL of 5.0 mM fresh prepared acetyl Coenzyme A solution in deionized water and 10 µL of cell lysate. The cuvette was pre-warmed to 25°C, and the reaction initiated by adding 10 µL of 0.3% (w/v) chloramphenicol solution. The increase in absorbance at 412 nm was recorded.

To calculate Units/mL in the lysate, the following equation was used where 0.2 is the total volume (in mL) of assay, df is the dilution factor, 0.0136 is the micromolar extinction coefficient for DTNB at 412 nm, and 0.01 is the volume of cell lysate used.

$$Units/mL\;CAT=\frac{\left(\Delta A_{412}/\min\;test-\Delta {\overset{–}{A}}_{412}/\min\;blank\;\left(0.2\right)\;\left(df\right)\right)}{\left(0.0136\right)\left(0.01\right)}$$

## 3 Results

### 3.1 Maximising transformation frequencies in *C. acetobutylicum*

**FIGURE 2:**
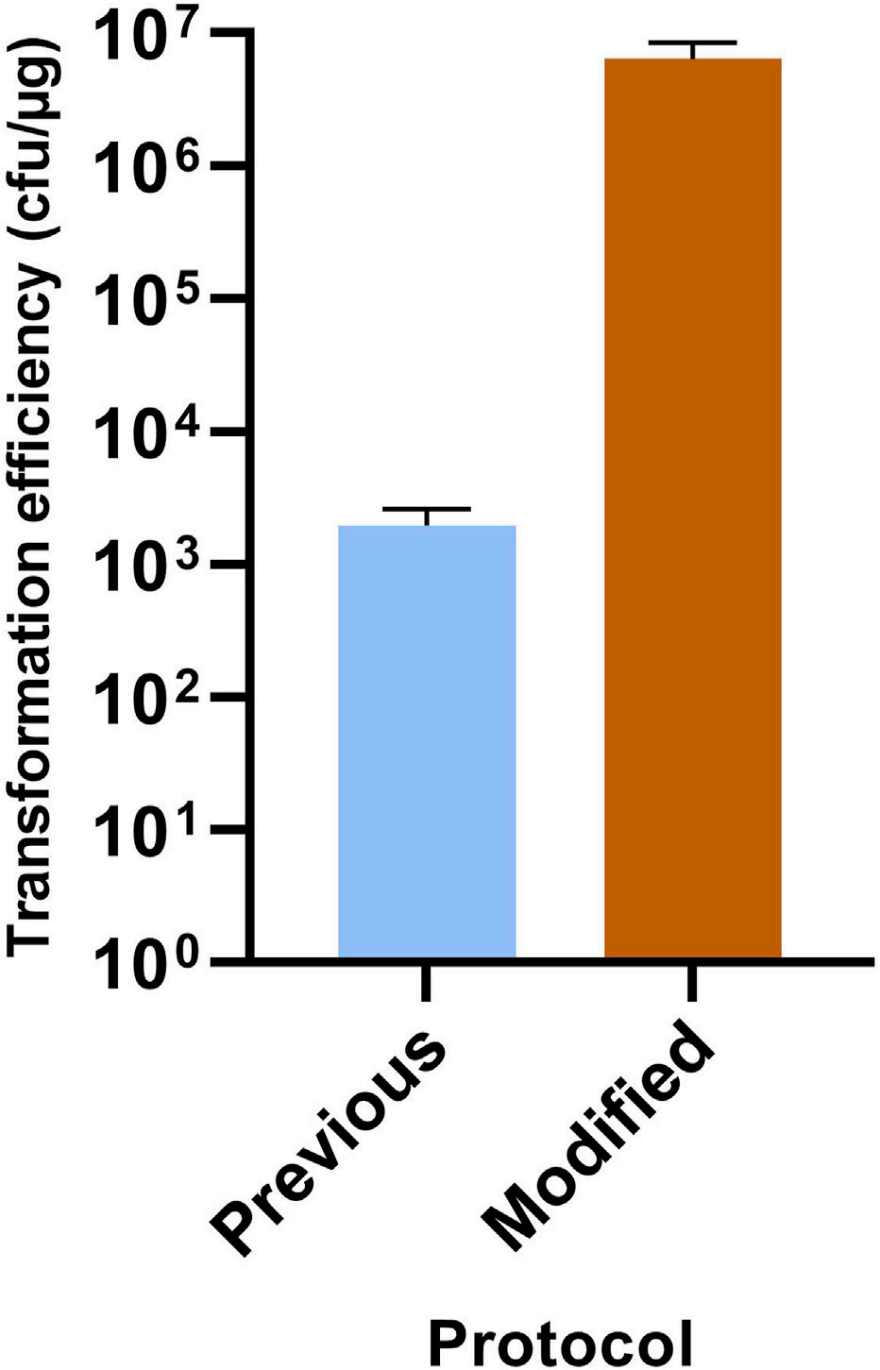
Improved transformation efficiency for *C. acetobutylicum.* Compared are the previously described protocol of Mermelstein et al. (1992) and the modified protocol described here. Experiments were undertaken in triplicate. Results are expressed as number of transformants per μg of plasmid pMTL85121 DNA.

As the envisaged knock-out strategy was based on the direct selection of double crossover mutants using suicide vectors, it was first necessary to optimize the transformation efficiency. A number of empirical alterations were therefore made to the originally described *C. acetobutylicum* transformation protocol (Mermelstein et al., 1992), and in particular to the steps involved in the preparation of competent cells. Similar to Herman et al. (2017), these included the addition of an extra EPB wash step and the extension of the 1–3 h recovery period after electroporation to at least 4 h. The principal difference between the two protocols, however, was that the vegetative cells for the preparation of competent cells were harvested at a much earlier phase of growth (OD600 of 0.2–0.25 as opposed to 1.1). The benefits of these modifications were demonstrated by transforming methylated pMTL851241 into competent *C. acetobutylicum* cells prepared by the two protocols. The Comparative transformation experiments with both methods were undertaken in triplicate and the average transformation efficiency in cfu per μg of plasmid shown to be 1.95E+03 ± 6.65E+02 and 6.41E+06 ± 2.04E+06 for the previous (Mermelstein et al., 1992) and the modified protocols, respectively (Figure 2). This represented a 3-log increase in transformation efficiency.

### 3.2 Exemplification and use of the knock-out (KO) system

When pMTL-KG146/7 plasmid derivatives carrying an appropriate mutant allele are transformed into the target clostridia, thiamphenicol (Tm) resistant (^R^) transformants can only arise if *catP* integrates into the genome following recombination between one or both of the plasmid-borne HAs and the equivalent region in the genome. Co-supplementation of media with 5FC ensures that the cells have lost the *codA-*bearing plasmid backbone and are, therefore, double crossover mutants. However, as with all insertional mutants of this type, the mutant generated is disadvantaged by the fact that the substantial size of the inserted marker (*pyrE::catP*) is likely to exert polar effects on flanking genes. Moreover, the same marker cannot be used again to generate a second mutation in the same cell. Fortunately, it is easily removed to generate a clean, in-frame deletion mutant by plating the Tm^R^, 5FC^R^ cells onto agar media lacking Tm but containing FOA. This selects for FOA^R^ cells in which *pyrE* (together with *catP*) has been excised due to resolution of the two directly repeated *dif*/*ran* sites to a single site by the hosts Xer site-specific recombinases (Bloor and Cranenburg, 2006). More importantly, the nature of the repeat sequences is such that an in-frame deletion of the targeted gene is formed—see Figure 1B.

In the initial experiments, KO plasmids targeting the three genes (*pheA, purD, argH*) were designed and built carrying appropriate homology arms and either the predicted *dif* sequence of *C. acetobutylicum* or a random 29 bp repeat sequence (*ran*) positioned at either end of the *catP-pyrE*. In all cases, using the new transformation procedure, between 1.0 and 2.0 × 10^1^ transformants were consistently obtained when plated on media supplemented with Tm. When plated on media additionally supplemented with 5FC, the numbers were slightly reduced, but in every instance several clones were routinely obtained. Moreover, in all cases, PCR screening indicated that the expected insertional mutant had been generated as a consequence of a double crossover integration.

Following their appearance, a representative Tm^R^ 5FC^R^ colony was restreaked onto CBM media supplemented with FOA and the supplement appropriate to the gene being targeted. These equated to phenylalanine in the case of *pheA,* arginine in the case of *argH* and a purine cocktail (adenine, guanine and hypoxanthine) in the case of *purD.* PCR screening of the FOA^R^ colonies obtained demonstrated that clones in which the *catP-pyrE* DNA segment had flipped out were always present and arose at an average frequency of 36% for the *dif* repeat sequence and 37% for *ran*, respectively.

Having generated the three double auxotrophic mutants, growth of each on P2 minimal media was shown (Supplementary Figure S2) to be restored through supplementation of the media with uracil and either phenylalanine (Δ*pyrE*-Δ*pheA* mutant)*,* purines (Δ*pyrE*-Δ*purD* mutant) or arginine (Δ*pyrE*-Δ*argH* mutant). Restoration of the mutants to prototrophy was also demonstrated by assembling specific ACE complementation vectors, equivalent to that previously described for the *pyrE* gene, plasmid pMTL-MEC6 (Ehsaan et al., 2016) and using ACE to replace each genomic mutant allele with a wildtype copy of the gene. This involved transforming a mutant (e.g., the Δ*pyrE*-Δ*pheA* mutant) with the requisite ACE complementation vector (e.g., pMTL-HZ1C), selecting a transformant colony on CGM media supplemented with thiamphenicol, and then streaking the colony onto P2 media supplemented with only uracil, and not, for instance, phenylalanine. In parallel, plasmid pMTL-JH14 (Heap et al., 2012) was used to restore the *pyrE* locus of each of the double mutants to uracil prototrophy, creating single mutants defective in *pheA, purD* and *argH.*

Finally, as the envisaged metabolic engineering strategy would require three mutant loci in the same cell, a triple auxotrophic mutant was made by knocking out the *purD* gene in the Δ*pyrE*Δ*pheA* double mutant and resultant triple auxotrophic mutant, Δ*pyrE*Δ*pheA*Δ*purD*, shown to be only capable of growth on P2 minimal medium supplemented with uracil, phenylalanine and the purine cocktail (Supplementary Figure S2).

### 3.3 Functionality of lactose inducible orthogonal expression system

In the current investigation we wished to explore the possibility of providing three different loci in the same cell to allow the stable, independent integration of different components of a metabolic pathway. Although the genes of a pathway may be disparately located, it is desirable that they are subject to common regulatory control. This may be achieved through the deployment of an orthogonal expression system (An and Chin, 2009; An and Chin, 2011; Costello and Badran, 2021). We have previously described a clostridial-derived orthogonal expression system (Zhang et al., 2015) based on the *Clostridioides difficile* (formerly *Clostridium difficile*) TcdR sigma factor that only recognises the promoter sequences of the *tcdA* and *tcdB* toxin genes (Dupuy et al., 2006). This was used to instigate transposition in *C.acetobutylicum* by placing the transposase of the *mariner* transposon used under the control of the P_*tcdB*_ promoter. The system was more recently refined in *C. autoethanogenum* (Woods et al., 2022) by placing expression of *tcdR* under the control of a lactose inducible system (*bgaR-P*_*bgaL*_) from *C. perfringens* (Hartman et al., 2011).

**FIGURE 3:**
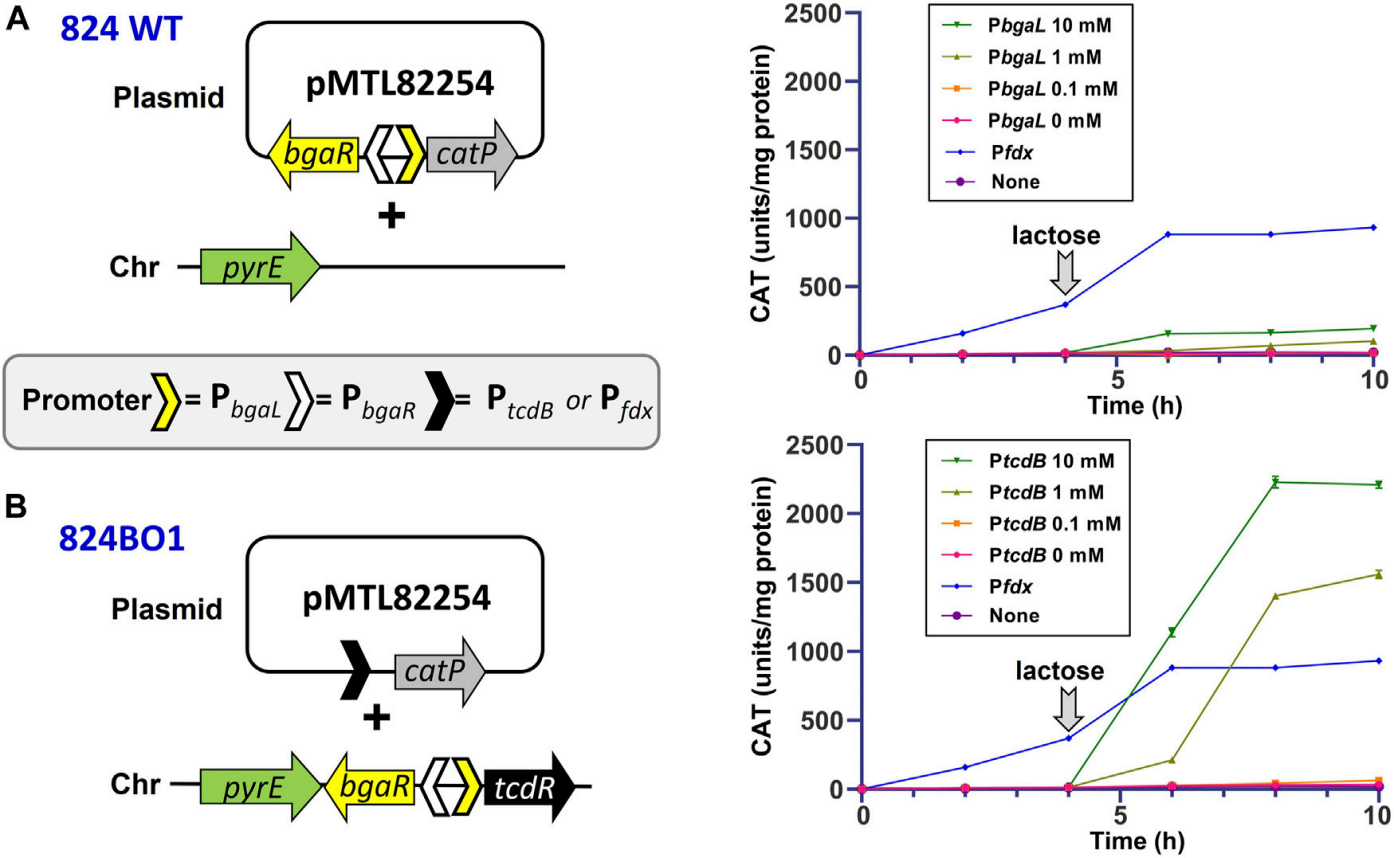
Controlled expression of the *catP* using a lactose inducible orthogonal TcdR system. The *C. acetobutylicum* hosts used were either the WT strain **(A)** or 824BO1 **(B)**, the latter of which carried an integrated copy of *tcdR* at the *pyrE* locus under the control of the BgaR::P_*bgaL*_ lactose inducible promoter system. Strain 824BO1 was transformed with derivatives of the reporter plasmid pMTL82254 (Heap et al., 2009) in which the *catP* gene was placed either under the control of the P_*tcdB*_ or P_*fdx*_ promoter. The WT was transformed with an equivalent plasmid but in which *catP* was under the control of *bgaR*::P_bgaL_. Cells were grown in 2YTG media and induced with lactose after 4 h (downward facing arrow) at concentrations of 0, 1.0, 1.0 and 10 mM. Also included was a *C. acetobutylicum* negative control carrying the reporter vector pMTL82254 alone with no promoter (None). Experiments were performed in triplicate.

To test the suitability of the lactose-induced orthogonal system in *C. acetobutylicum,* the *bgaR-P*_*bgaL*_::*tcdR* expression cassette described by Woods et al. (2022) was integrated into the *pyrE* locus of *C. acetobutylicum* following its subcloning into pMTL-ME6C. The isolated strain was designated 824BO1. In parallel, the three promoter P_*tcdB*_, P_*fdx*_ and *P*_*bgaL*_, the latter in combination with *bgaR*, were inserted into the modular reporter vector pMTL82254 (Heap et al., 2009) to give plasmids pMTL82254::PtcdB, pMTL82254::Pfdx and pMTL82254::PbgaL, respectively (Figure 3). The former two plasmids were transformed into the *C. acetobutylicum* strain 824BO1, while the latter was transferred into the 824 WT strain. Each of the resultant transformants clones were cultivated in 2YTG medium and the level of CAT activity present in the cell lysates of harvested cells measured periodically. Replicate cultures of those cells carrying the *bgaR-P*_*bgaL*_ inducible system were induced with lactose 4 h after inoculation. In the case of WT cells carrying plasmid pMTL82254::PbgaL, the level of CAT activity some 6 h after induction with 1 and 10 mM lactose equated to 102 and 195 units/mg protein, respectively. In contrast, the levels in 824BO1, in which *catP* expression was indirectly regulated by lactose via the TcdR-based orthogonal system, were over 10-fold higher. Thus, CAT activity 6 h after induction with either 1 or 10 mM lactose corresponded to 1,560 and 2,209 units/mg protein, respectively (Figure 3). The level of expression achieved under both induction conditions were greater than that seen in cells carrying the plasmid (pMTL82254::Pfdx) in which *catP* was under the control of the strong P_*fdx*_ promoter, where CAT levels reached 933 units mg/protein.

### 3.4 Generation of acetone and isopropanol producing strains in the triple auxotroph

**FIGURE 4:**
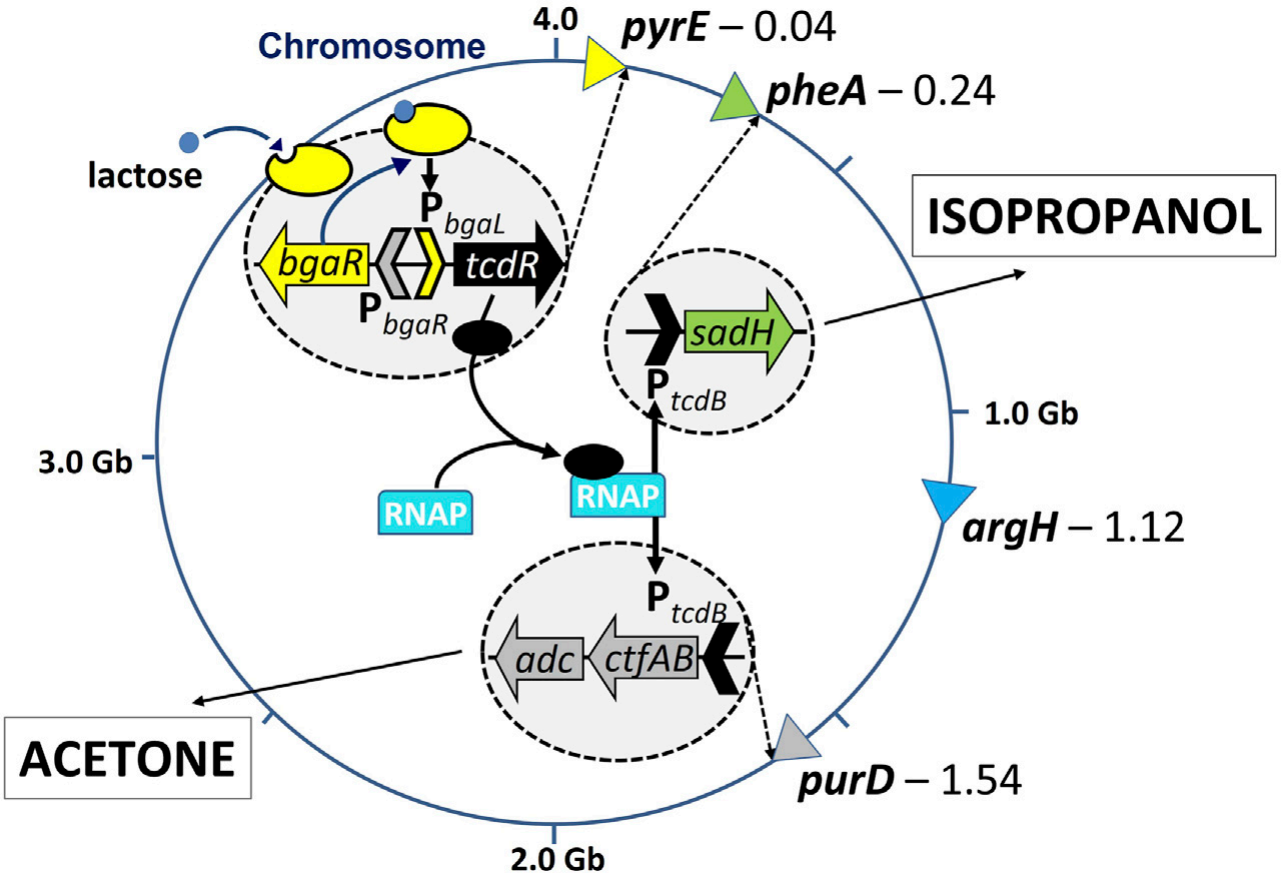
Acetone and isopropanol producing *C. acetobutylicum* strain; bearing *bgaR-P*_*bgaL*_::*tcdR* (lactose inducible *tcdR*) at *pyrE* locus, *ctfA/B* and *adc* (encoding CoA transferase and acetoacetate decarboxylase) at *purD* locus and *sadh* (encoding a secondary alcohol, dehydrogenase, sADH) at *pheA* locus. The position of the four loci on the 4.0 Gb *C. acetobutylicum* chromosome is shown. The *bgaR* gene encodes the BgaR response regulator which, following binding of lactose brings about transcription of the *tcdR* gene. The TcdR sigma factor produced promoters binding of RNA polymerase (RNP) to the P_*tcdB*_ promoter upstream of *sadh* at the *pheA* locus and *ctfA/B*:*adc* at the *purD* locus resulting in production of isopropanol and acetone, respectively.

In order to test the utility of multiply marked auxotrophic mutants, we chose to integrate the genes necessary for production of acetone (*ctfA/B* and *adc*, encoding CoA transferase and acetoacetate decarboxylase, respectively) and isopropanol (*sadh*, codes for a secondary alcohol dehydrogenase, from *Clostridium beijerinckii*) at two different loci and to place them both under the control of the P_*tcdB*_ promoter. Transcription from both copies of this promoter would be mediated by TcdR produced from the *tcdR* gene integrated at the *pyrE* locus and under the control of the lactose inducible P_*bgaL*_ promoter (Figure 4). As the proposed strategy required just three loci, only *pyrE, pheA* and *purD* were selected and an appropriate triple auxotrophic mutant made carrying all three deletions. Those genes necessary for the production of acetone (P_*tcdb*_::*ctfA/B*-*adc*) were integrated at the *purD* locus using the ACE PurD complementation vector pMTL-BO1C, whereas the gene required for production of isopropanol (P_*tcdB*_::*sadh*) was inserted at the *pheA* locus using the ACE PheA complementation vector, pMTL-HZ1C. This led to the creation of three strains: i) 824BO1, carrying *tcdR* at the *pyrE* locus; ii) 824BO2, as 824BO1 but with the acetone operon at the *purD* locus, and; iii) 824BO3, as 824BO2, but with the secondary alcohol dehydrogenase at the *pheA* locus. The *pheA* and *purD* loci of strain 824BO1, and the *pheA* locus of 824BO2, were all converted back to wildtype using the appropriate ACE correction vector prior to the analysis of the strains in fermenters. The result of phenotypic analysis of the strains generated is shown in Supplementary Figure S3.

### 3.5 Acetone and isopropanol-butanol-ethanol (IBE) production

**FIGURE 5:**
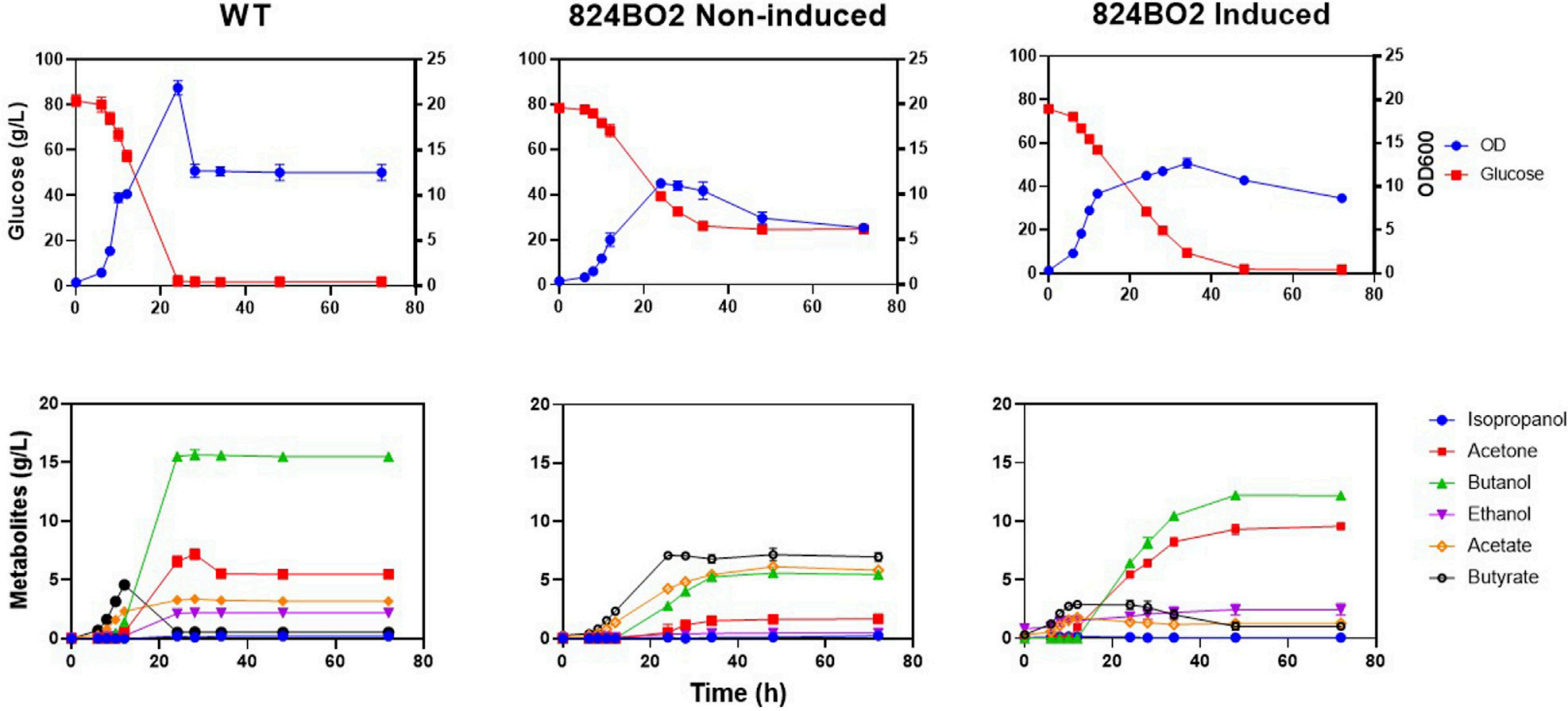
Lactose induced acetone production. *C. acetobutylicum* strain 824BO2 and the wild type ATCC 824 control were grown in CGM under pH-control (≥5.0). Cultures were induced with lactose at 6 h. Growth and metabolic profiles, with and without 10 mM lactose induction, are shown. Experiments were carried out in triplicate and time points are given as average values.

To investigate the impact of the integrated acetone operon in *C. acetobutylicum,* the solvent profile of strain 824BO2 was analysed. Up to 9.31 ± 0.30 g/L acetone was produced with a 10 mM lactose induction while the wild type *C. acetobutylicum* 824 produced only 5.48 ± 0.03 g/L (Table 2). An increase in ethanol (2.43 ± 0.33 g/L) (Figure 5) compared to wild type (2.20 ± 0.01 g/L) was also observed while butanol was higher in the WT (15.50 ± 0.25 g/L) than in 824BO2 (12.23 ± 0.20 g/L). At the same time, strain 824BO2 exhibited a decrease in acid production.

**TABLE 2:** Summary of the IBE production achieved here and in comparable studies.

Study	IPA (g/L)	BuOH (g/L)	EtOH (g/L)	IBE (g/L)	Gene Location^b^	Strain^a^	IBE gene promoters			pH control	Comment
							*sadh*	*ctfA/B*	*adc*		
This study	4.40	13.49	3.09	19.80	Chr	ATCC 824	P_tcdB_	P_tcdB_	P_tcdB_	Yes	*tcdR* integrated downstream of *pyrE* under control of P_bgl_ *, ctfA/B-adc* and *sadh* integrated downstream of *purD* and *pheA*, respectively, under control of P_tcdB_
						*bgaR::tcdR*					
						integrant					
Banker et al. (2015)	2.51	10.78	2.0	15.30	Chr	DSM 792	P_adc_	native	native	Yes	*sadh* under P_adc_ promoter integrated in Chr, *ctfA/B-adc* genes on the pSOL megaplasmid
						wildtype					
Wang et al. (2018)	6.62	10.51	1.24	17.77	pIMP1	XY16	P_ptb_	native	native	Yes	*sadh* expressed from P_ptb_ on multicopy plasmid, *ctfA/B-adc* on megaplasmid. Regulation of intracellular NAD(P)H levels by CaCO_3._
					(pIM13)	wildtype					
Dusseaux et al. (2013)	4.22	13.8	0.46	18.48	pCLF942	ATCC 824	P_ptb_	P_ptb_	P_ptb_	Yes	*Δbuk* made in a *Δcac15Δupp* mutant. *ctfA/B-sadh-adc* expressed from P_ptb_ on a multicopy plasmid.
					(pIM13)	*Δbuk*					
Dusseaux et al. (2013)	4.75	14.63	1.01	20.40	pCLF952	ATCC 824 Δ*buk*	P_thl_	P_thl_	P_thl_	Yes	*Δbuk* made in a *Δcac15Δupp* mutant. *ctfA/B-sadh-adc* expressed from P_thl_ on a multicopy plasmid.
					(pIM13)						
Jang et al. (2013)	3.60	14.80	9.5	27.90	pIPA100	ATCC 824 (BKM19)	P_thl_	native	native	Yes	BKM19 (hyper-ABE producer) *buk*-mutant carrying s*adh*-*hydG* expressed from P_thl_ on multicopy plasmid.
					(pIM13)						
Dai et al. (2012)	7.60	15.00	1.28	23.88	pIMP1	DSM 1731 (Rh8)	P_thl_	native	native	Yes	Rh8 (butanol tolerant mutant) carries P_*thl*_ *-sadh* on a pIMP1 and *ctfA/B-adc* on the pSMBa megaplasmid.
					(pIM13)						
Collas et al. (2012)	8.80	13.70	1.5	24.00	pFC007	ATCC 824	P_thl_	P_thl_	P_thl_	No	harbouring P_*thl*_ -*sadh-ctfA/B-adc* genes expressed from P_*thl*_ on multicopy plasmid based on pAMβl
					(pAMβl)	wildtype					
Lee et al. (2012)	5.10	8.00	0.80	13.90	pIPA3	ATCC 824	P_adc_	P_adc_	P_adc_	No	*ctfA/B-adc-sadh* under control of P_adc_ promoter on multicopy plasmid—batch fermentation in flasks.
					(pIM13)	wildtype					
Lee et al. (2012)	6.10	10.20	0.80	17.10	pIPA3	ATCC 824	P_adc_	P_adc_	P_adc_	Yes	*ctfA/B-adc-sadh* under control of P_adc_ promoter on multicopy plasmid—in a fermentator.
					(pIM13)	wildtype					
Lee et al. (2012)	4.40	14.10	1.90	20.40	pIPA3	ATCC 824	P_adc_	P_adc_	P_adc_	Yes	As above but undertaken in a *buk-* mutant as opposed to the wildtype.
					(pIM13)	*buk* ^-^					

^a^ All strains described in Table 2 are *C. acetobutylicum* carrying the megaplasmid pSOL, or equivalent.

^b^ Chr indicates chromosome.

**FIGURE 6:**
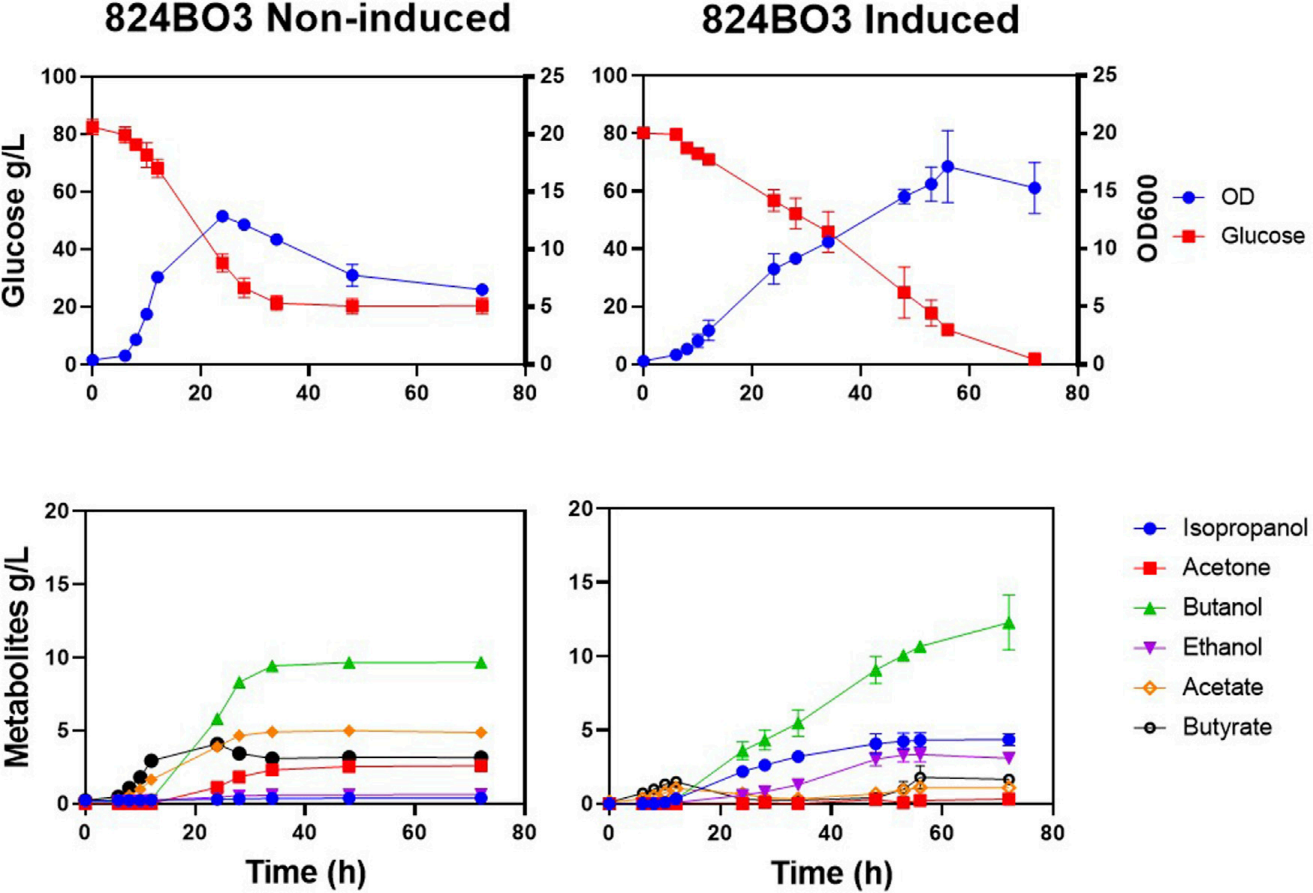
Lactose induced isopropanol production. *C. acetobutylicum* strain 824BO3 was cultivated under pH-control (≥5.0) CGM and induced with lactose at 6 h. Growth and metabolic profiles, with and without 10 mM lactose induction, are shown. Experiments were carried out in triplicate and time points are given as average values.

Having shown that up to 41% more acetone was produced by the integrated acetone operon in strain 824BO2, the production of IBE from 824BO3, containing the *sadh* gene was investigated. Fermentation was carried out in bioreactors with pH maintained at ≥ 5.0. Strain 824BO3 had a longer lag phase and complete glucose consumption was observed at 72 h compared with the WT which had completely used up glucose by 24 h. With induction, up to 4.4 g/L isopropanol was produced at 72 h (Figure 6) with residual acetone of 0.32 ± 0.06 g/L. As expected, less acetone was produced than by the WT due to its conversion to isopropanol by the sADH. As expected, in the absence of lactose induction, little or no isopropanol (0.39 ± 0.00 g/L) production was observed.

Similar to strain 824BO2, more ethanol (3.09 ± 0.11 g/L) and less butanol (12.30 ± 1.31 g/L) was produced compared with the WT which made 2.20 ± 0.01 g/L and 15.50 ± 0.25 g/L ethanol and butanol, respectively. Strain 824BO3 also produced less acids than the WT (Figure 5). The non-induced 824BO3 strain showed incomplete glucose utilization, accumulation of residual acids and a decrease in solvent concentration. Table 2 summarises the IBE production in *C. acetobutylicum* from previous researchers using plasmid borne as well as chromosomally integrated heterologous genes.

## 4 Discussion

In the current study we used an inducible orthogonal system to control the disparately located genes responsible for acetone and isopropanol production. In the strain engineered for just acetone production, 824BO2, the decrease in acid production compared to the WT was consistent with the fact that with the onset of solventogenesis, acetate and butyrate are re-assimilated and metabolized concomitantly with glucose. Acid uptake is via the acetoacetyl-CoA:acyl-CoA transferaseA/B (*ctfA/B*) with acetoacetyl-CoA serving as the CoA donor (Lütke-Eversloh and Bahl, 2011) and this re-assimilation has been directly coupled with the formation of acetone (Jones and Woods, 1986).

Acetone is formed in two steps from acetoacetyl-CoA; CoA is first removed by CtfA/B yielding acetoacetate which is subsequently decarboxylated by ADC to form acetone. The increase in ethanol could be from the additional acetyl-CoA made available from the uptake of acetate as a consequence of the overexpression of the integrated *ctfA/B* genes. A similar increase in total solvents and decrease in residual carboxylic acids in *C. acetobutylicum* has previously been reported following overexpression of the genes of the acetone operon (Mermelstein and Papoutsakis, 1993; Jang et al., 2012). The metabolic profile of 824BO2 was strongly influenced by the lactose induced overexpression of the acetone operon integrated in the genome, as was evident from the profile of the uninduced cultures (Figure 5). In this instance, the consumption of glucose was incomplete, more acid was accumulated and solvent yields were lower.

Converting acetone to isopropanol results in a mixture of isopropanol, butanol and ethanol, commonly referred to as IBE. This mix is more attractive for fuel applications than ABE as it can be used directly (Collas et al., 2012; Li et al., 2019). Indeed, its use as a fuel additive in the production of gasoline or diesel oil has already been reported (Peralta-Yahya and Keasling, 2010; Li et al., 2019). Here, the total IBE produced by strain 824BO3 from 80 g/L glucose was 19.8 g/L. In comparison with *Clostridium beijerinckii*, a natural producer of IBE, these yields are higher. In 2000, Shaheen and co-workers (Shaheen et al., 2000) undertook some comparative fermentations of industrial clostridial strains and demonstrated that *C. beijerinckii* NRRL B592 produced 16.2 g/L total solvents from maize mash (80 g/L). Survase et al. (2011) reported a batch production of 5.9 g/L IBE (3.7 g/L butanol and 2.2 g/L isopropanol) while continuous fermentation yielded 7.51 g/L. In a more recent study, *C. beijerinckii* BGS1 was reported to produce 13.6 g/L IBE (10.2 g/L butanol and 3.4 g/L isopropanol) with negligible ethanol from 60 g/L glucose (Zhang et al., 2018).

Previously described engineering of *C. acetobutylicum* for IBE production have largely relied on the use of high copy number autonomous plasmids requiring antibiotic maintenance. However, while the incorporation of operons into autonomous plasmids has the advantage of higher copy number, guaranteeing higher yields of products, the plasmids are invariably segregationally unstable and may be lost from the population. Reduced productivity due to plasmid loss constitutes a major industrial problem (Friehs, 2004; Peubez et al., 2010; Seiben et al., 2016; Fedorec et al., 2019). Although this can be avoided by endowing the plasmid antibiotic resistance genes and including the requisite antibiotic in the media, such measures are unwise if the spread of antimicrobial resistance and the associated environmental and health problems (Hagg et al., 2004) are to be avoided. Moreover, the addition of antibiotics to cultures is both expensive and undesirable (Kroll et al., 2010). Heterologous genes can be stabilized by chromosomal integration (Kroll et al., 2010).

Integration of gene sets into the chromosome overcomes these drawbacks, producing strains that are segregationally stable and more suitable for industrial applications. Banker et al. (2015), for instance integrated the *sadh* gene under the control of the P_*adc*_ promoter into the chromosome of *C. acetobutylicum* using allelic exchange. The engineered strain was able to produce 2.51 g/L isopropanol and 18 g/L IBE, representing the highest reported yield of isopropanol and IBE from a chromosomally engineered *Clostridium*. In the present study the use of the lactose inducible TcdR system, allowed this benchmark to be exceeded, achieving 4.4 g/L isopropanol and 19.8 g/L IBE. These levels were reached through the addition of high concentrations of the lactose inducer, which lead to elevated production of the TcdR sigma factor. Whilst the use of such an inducible system is probably not applicable on an industrial scale, the outcome has demonstrated both the feasibility of controlling the expression of chromosomally disparate gene sets with the same orthogonal sigma factor and given an indication of the level of expression required.

It was noticeable that 824BO2/3 produced slightly reduced levels of solvents under non-induced conditions. As the parental auxotrophic mutants were unaffected in solvent production (data not shown), the likely cause of this phenotype was the presence of *tcdR* in the *pyrE* locus. Although the *C.acetobutylicum* genome carries no sequences that closely resemble the *tcdA* or *tcdB* promoters, non-specific binding of the TcdR to the chromosome or pSOL megaplasmid that effect the expression of genes involved in solvent production cannot be ruled out. However, whatever the basis of the any hypothetical interaction(s), it did not prevent the attainment of high levels of solvents following lactose-induced overproduction of TcdR.

The mutational tool used to create the suite of auxotrophic mutants used here provides a simple and reliable route to mutant acquisition for strategies reliant on the counter-selection markers *codA* or *pyrE* (Minton et al., 2016). This is because, although two distinct steps are involved, positive selection of the desired outcome is possible at both stages. Thus, the double crossover mutant is directly selected in the first step and the clean, in-frame deletion mutant at the subsequent second step. In contrast, previously developed methods first select single crossover integrants before selecting clonal populations in which an excision event has occurred by plating on media containing the counter-selection agent. At this second stage, both wildtype ‘revertants’ and double cross-over mutants are present, necessitating the use of an additional screen (PCR) to identify the in-frame deletion mutant clones. While the method requires the starting strain to be a *pyrE* mutant, these are relatively easily generated using ACE or a RAM-less version of the ClosTron (Minton et al., 2016).

Although in recent years, CRISPR/Cas9 systems have largely replaced the use of counter-selection markers for mutant generation, they are not without problems. Cas9 has off-target effects, distributed license agreements for its use are expensive and the long-lasting patent battle between the two major players has caused uncertainty with potential licensees. In contrast, for commercially orientated studies, the system described here is unfettered by licensing issues.

## 5 Conclusion

A system has been devised that allows the introduction of different components of a metabolic pathway at distinct loci around the *C. acetobutylicum* chromosome, based on the creation of multiple auxotrophic mutant alleles using a novel procedure that facilitates the direct selection of deletion mutants. Components can be rapidly integrated using allele-coupled exchange (ACE) and conveniently selected on the basis of the restoration of prototrophy. Our experiments demonstrate that the orthogonal sigma factor TcdR, following integration of its encoding gene at one locus, can be used to simultaneously control different gene sets integrated at distant chromosomal loci. As these gene sets have disparate locations, recombination between the identical promoters is not an issue as may be the case when tandemly arranged. By placing the production of TcdR under the control of the lactose inducible BgaR::P_*bgaL*_ promoter system we established that *catP* expression from the P_*tcdB*_ promoter when induced with 1–10 mM lactose was over 10-fold higher than if *catP* was directly under the control of BgaR::P_*bgaL*_. The system was capable of producing 4.4 g/L isopropanol and 19.8 g/L IBE.

## Data Availability

The original contributions presented in the study are included in the article/Supplementary Material, further inquiries can be directed to the corresponding author.
