# Supplementary material for: Chromosomal engineering of inducible isopropanol- butanol-ethanol production in Clostridium acetobutylicum
Source: Front Bioeng Biotechnol. 2023 Jun 16;11:1218099. doi: 10.3389/fbioe.2023.1218099 (PMC10312008; doi:10.3389/fbioe.2023.1218099)
Supplement: Supplementary file 1 [file DataSheet1.docx]

***Supplementary Material***

**Chromosomal Engineering of Isopropanol-Butanol-Ethanol production in *Clostridium acetobutylicum***

Bunmi B Omorotionmwan^a, #^, Hengzheng Wang^a^, Jonathan P Baker^a^, Krzysztof Gizynski, Minyeong Yoo^a^, Ying Zhang^a^, Nigel P Minton^a,b^*

^a^ *Clostridia Research Group, BBSRC/EPSRC Synthetic Biology Research Centre (SBRC), Biodiscovery Institute, School of Life Sciences, The University of Nottingham, Nottingham, NG7 2RD, United Kingdom*

*^b^ NIHR Nottingham Biomedical Research Centre, Nottingham University Hospitals NHS Trust and the University of Nottingham, Nottingham, United Kingdom*

*^#^ Current address: Biosciences Department, School of Science and Technology, Nottingham Trent University, Clifton Campus, Nottingham, NG11 8NS*

*** Correspondence:** Corresponding Author: nigel.minton@nottingham.ac.uk

**Key words**: Auxotrophic mutant, Orthogonal, Inducible system, Isopropanol-Butanol-Ethanol, *Clostridium,* allele-couple exchange

# Supplementary Data

**PLASMID CONSTRUCTION**

**pMTL-KG146 and pMTL-KG147 Knock-out Vectors**

To make the knock-out vectors pMTL-KG146 and pMTL-KG147, Gibson assembly was used. Firstly, primers codA_F, codA_R, colE1_F and colE1_R were used to amplify catP, codA and colE1 from plasmid pMTL_SC7515 (Cartman *et al*, 2012) with Q5 polymerase; primers catP_F1 (dif), catP_F2 (ran), catP_R, pyrE_F, pyrE_R1 (dif) and pyrE_R2 (ran) were used to amplify catP and pyrE from pMTL_ME6 (Ehsaan *et al*, 2016). All PCR fragments were assembled in a four-fragment Gibson assembly. This resulted in pMTL-KG146 and pMTL-KG147 which were transformed in *E. coli* DH5α using heat shock according to New England Biolabs (NEB) instructions. After growth of colonies, PCR screening and miniprep, the correct plasmids were used for the construction of knock-out vectors as below.

A 1000bp LHA to mediate the truncation of 405 bp from the 3‘-end of the *purD* gene was PCR amplified from *C. acetobutylicum* genomic DNA using oligonucleotides purD_LHA_MreI_F and purD_LHA_FspAI_R flanked by *Mre*I and *Fsp*AI restriction recognition sites. The RHA was an amplified 1000bp region immediately downstream of *purD* gene using oligo purD_RHA_SgrDI_F and purD_RHA_MauBI_R flanked by *Sgr*DI and *Mau*BI restriction recognition sites. The LHA and RHA were cloned into pMTL-KG146 or pMTL-KG147 following their sequential cleavage with *Mre*I/*Fsp*AI and *Sgr*DI/*Mau*BI respectively. This resulted in the generation of pMTL-KG146_purD and pMTL-KG147_purD. Similar procedure was followed in generating the knock-out plasmids for argH (pMTL-KG146_argH and pMTL-KG147_argH), and pheA (pMTL-KG146_pheA and pMTL-KG147_pheA) using the oligonucleotides listed in table S1.

**Complementation vectors**

To generate equivalent complementation vectors to pMTL-ME6C, restriction endonuclease cloning was utilized. Oligonucleotides purD_cLHA_SbfI_F and purD_cLHA_NotI_R flanked by *Sbf*I and *Not*I restriction recognition sites were used to amplify *purD* region from *C. acetobutylicum* genomic DNA using Q5 polymerase. This encompassed the 300 bp SHA as well as the deleted *purD* region to be repaired in the mutant. The LHA (encompassing 1200bp downstream of *purD*) was similarly PCR amplified from *C. acetobutylicum* genomic DNA using purD_cRHA_NheI_F purD_cRHA_AscI_R flanked by *Nde*I and *Asc*I sites. The SHA and LHA were cloned following their appropriate sequential cleavage with *Sbf*I/*Not*I and *Nde*I/*Asc*I. This resulted invector pMTL-BO1C. A similar procedure was followed in creating the complementation vectors pMTL-HZ1C and pMTL-HZ2C using oligonucleotides listed in table S2.

# Supplementary Figures and Tables

## Supplementary Figures


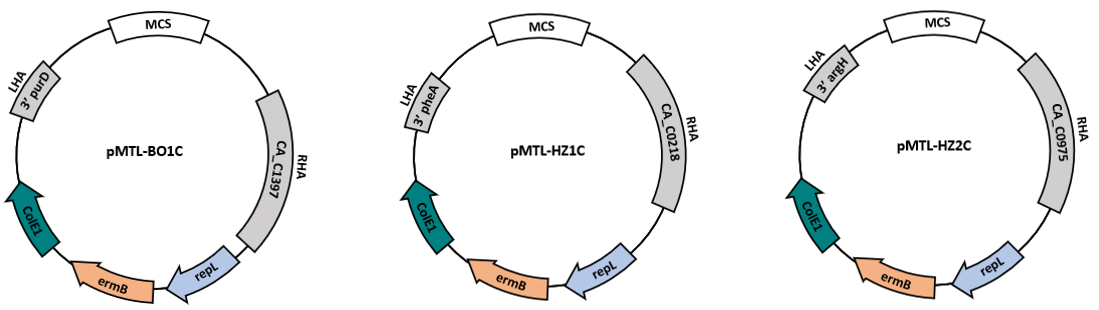


**Figure S1: ACE complementation vectors pMTL-BO1C (*purD*), pMTL-HZ1C (*pheA*) and pMTL-HZ2C (*argH*).**

**
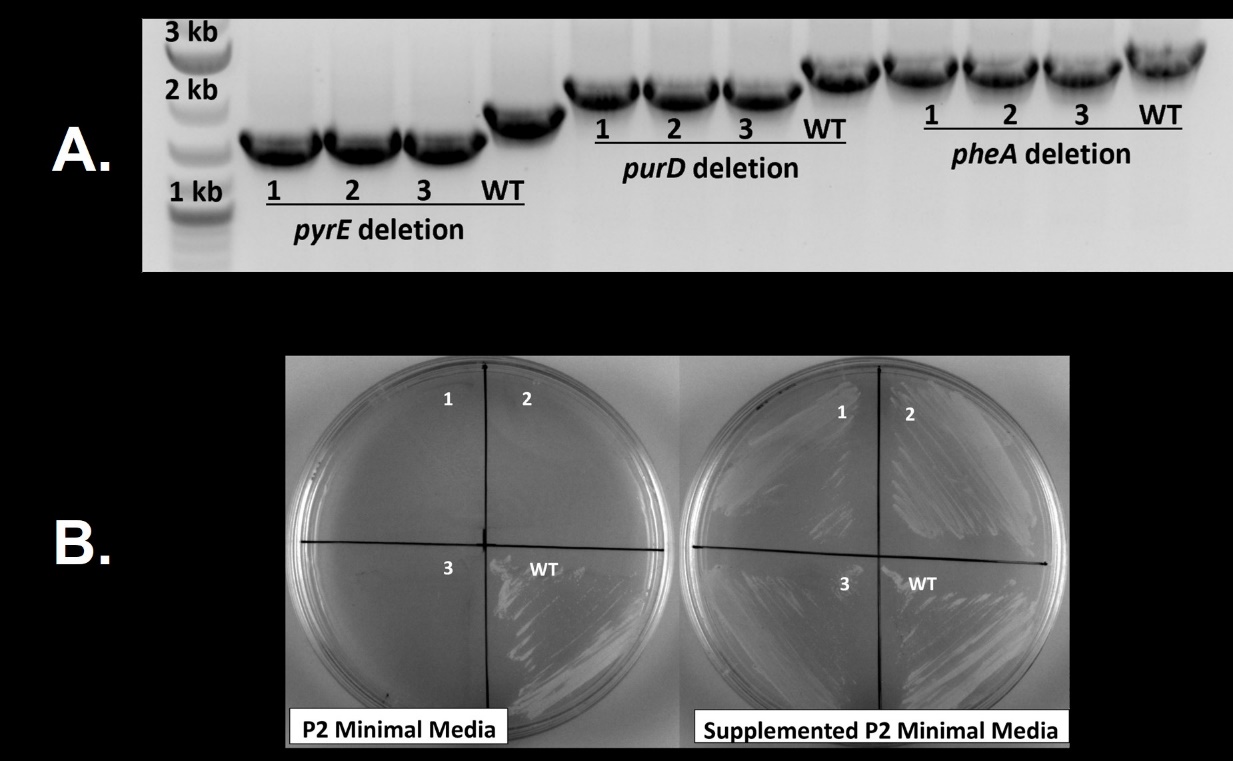
**

**Figure S2. Creation of the triple auxotrophic mutant of *C. acetobutylicum.*** (**A)** PCR screening of Δ*pyrE*Δ*pheA*Δ*purD* using flanking primers for or all three loci. For the *pyrE* deletion, flanking primers were JH14F/R which generates a DNA fragment of 1686 bp compared to 1989 bp in the WT. In the case of the *purD* deletion, the flanking PCR primers purD_ch_F/R generated a 2282 bp DNA fragment with the mutant compared to 2687 bp with the WT. Finally, for the *pheA* deletion the pheA_ch_F/R flanking PCR primers generated a 2819 bp fragment with the mutant and 3090 bp fragment for the WT. (B**)**: *C. acetobutylicum* Δ*pyrE*Δ*pheA*Δ*purD* (1,2,3) are only able to grow on supplemented P2 minimal medium while WT grows on both supplemented and non- supplemented minimal medium


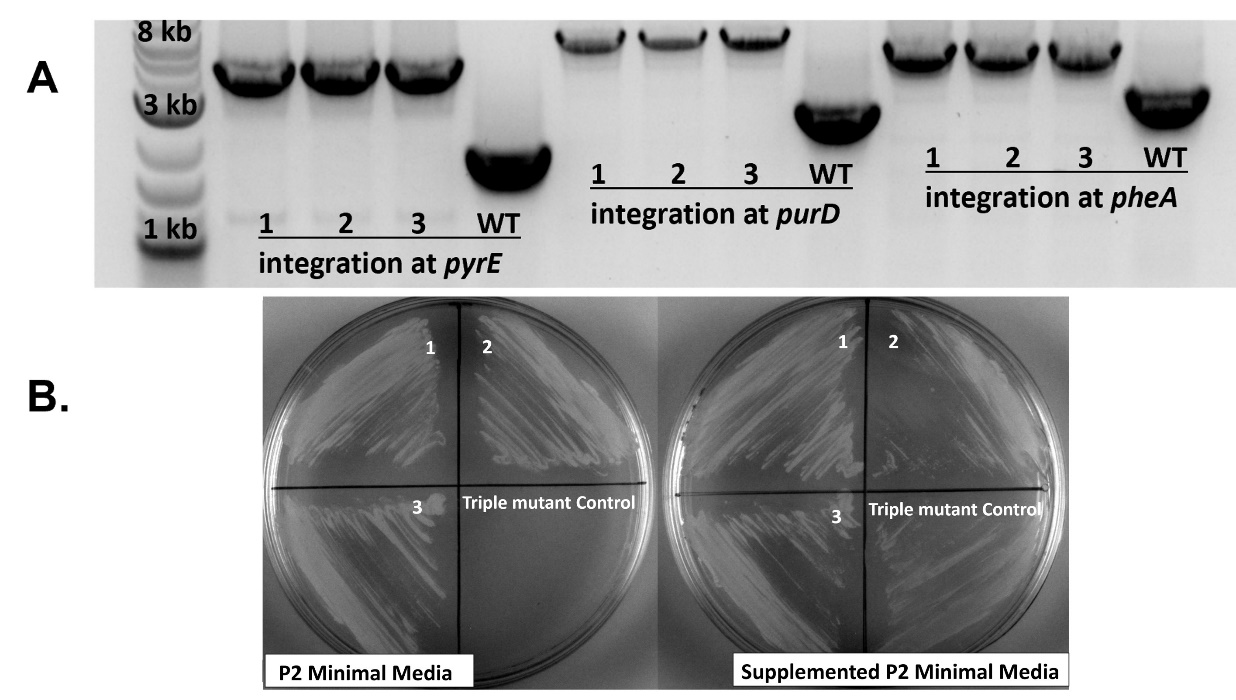


**Figure S3. Gene integration at multiple genomic loci**. **A.** PCR screening of final integrated strain, 824BO3 (1,2,3) with the lactose inducible *tcdR* system integrated at *pyrE* locus, acetone operon integrated at the *purD* locus and secondary dehydrogenase integrated at the *pheA* locus. At *pyrE*, flanking primers were JH14F/R, yielding a WT DNA band of 2687 bp and a 4268 bp DNA band in the integrant; at *purD*, flanking primers were purD_ch_F/R, yielding a WT DNA band of 1989 bp and a 5230 bp DNA band in the integrant, and; for *pheA*, flanking primers were pheA_ch_F/R, yielding A WT DNA band of 3090 bp and a 6700 bp DNA band in the integrant. **B**: 824BO3 (1,2,3) are able to grow on both supplemented and non- supplemented P2 minimal medium while the parent strain, the triple auxotrophic (Δ*pyrE*Δ*pheA*Δ*purD*) *C. acetobutylicum* mutant, cannot grow without supplementation.

## Supplementary Tables

**Table S1. List of Oligonucleotides**

| **Primer** | **Sequence (5′→ 3′)** | **Template/Purpose** |
| --- | --- | --- |
| JH14/F | TAGCACAATTGTATTTGGACTTCTTTAAATAAAAACATGG | Flanking primers for *pyrE* locus (F) |
| JH14/R | TTGATGATGTTTGTCTTGATGACTCAACATGC | Flanking primers for *pyrE* locus (R) |
| colE1_F | ACCGTCGACGATCGCGCGCGTCCTTTTTG | Forward primer for ColE1 fragment in Gibson Assembly |
| colE1_R | CTTCTATAACTTTATCTATATTTAAAGAATATGCATCATATACTATCTCGATCCG | Reverse primer for ColE1 fragment in Gibson Assembly |
| codA_F | TATAGATAAAGTTATAGAAGCAATAGAAGATTTAG | Forward primer for *codA* fragment in Gibson Assembly |
| codA_R | TGATGCGCATTACGCCGGCGATGAATTC | Reverse primer for *codA* fragment in Gibson Assembly |
| catP_F1 (dif) | CGCCGGCGTAATGCGCATCAGCTATCTCAGATACGATCGATTACAACAGTGGGCAAGTTGAAAAATTCACAAAAATGTGGTATAAT | Forward primer for catP fragment with *dif* site in Gibson Assembly |
| catP_F2 (ran) | CGCCGGCGTAATGCGCATGAAGACTATAATGGATATTATGTTAAATAGTGGGCAAGTTGAAAAATTCACAAAAATGTGGTATAAT | Forward primer for *catP* fragment with *ran* site in Gibson Assembly |
| catP_R | TTTATATCTTCTCCTTAGTTACTATTTATCAATTCC | Reverse primer for *catP* fragment in Gibson Assembly |
| pyrE_F | AACTAAGGAGAAGATATAAATGAGTAATATAAATGTTATAGATATATTAAAAGAATC | Forward primer for *pyrE* fragment in Gibson Assembly |
| pyrE_R1 (dif) | CGCGCGCGATCGTCGACGGTTGTAATCGATCGTATCTGAGATAGCTGTTAGGGTAACAAAAAACACCGTATTTCTACGA | Reverse primer for *pyrE* fragment with *dif* site in Gibson Assembly |
| pyrE_R2 (ran) | CGCGCGCGATCGTCGACGGTTGTATTTAACATAATATCCATTATAGTCTTCTTAGGGTAACAAAAAACACCGTATTTCTACGA | Reverse primer for *pyrE* fragment with *ran* site in Gibson Assembly |
| *argH*-ch-F | TGATGTTGAGGTAATAGCTGTATG | Flanking primers for *argH* locus (F) |
| *argH*-ch-R | GTGTATTCTCTATATTATCTCTTGC | Flanking primers for *argH* locus (R) |
| pheA_ch_F | CATGCAGATATATACATTCCTCTG | Flanking primers for *pheA* locus (F) |
| pheA_ch_R | CTCATTGACTTATAATCAAAACGA | Flanking primers for *pheA* locus (R) |
| purD_ch_F | TATGGAATTTCAATGGTTATGACAGG | Flanking primers for *purD* locus (F) |
| purD_ch_F | ATCACCCTCCATAGTAGTTTCC | Flanking primers for *purD* locus (R) |
| argH_LHA_MreI_F | GCTGGC**CGCCGGCG**GATTTATATGATCACAAAGA | Forward primer to clone in LHA into *argH* knock-out vector |
| argH_LHA_FspAI_R | GGCGTC**ATGCGCAT**GCTATTAAATCACCATAAAC | Reverse primer to clone in LHA into *argH* knock-out vector |
| argH_RHA_SgrDI_F | GCTGGC**CGTCGACG**TAAAGCATAGGGTTTTCTTAGGTATC | Forward primer to clone in RHA into *argH* knock-out vector |
| argH_RHA_MauBI_R | GGCGTC**CGCGCGCG**TGCAAGAAGTGTTCCTATTTTTG | Reverse primer to clone in RHA into *argH* knock-out vector |
| pheA_LHA_MreI_F | GCTGGC**CGCCGGCG**GCATAGACAAAGAAGTGTAT | Forward primer to clone in LHA into *pheA* knock-out vector |
| pheA_LHA_FspAI_R | GGCGTC**ATGCGCAT**GAATCTTGTCTCATTTCCCT | Reverse primer to clone in LHA into *pheA* knock-out vector |
| pheA_RHA_SgrDI_F | GCTGGC**CGTCGACG**TAAAAAATTATTTTGCCTTAAAGGAATAC | Foward primer to clone in RHA into *pheA* knock-out vector |
| pheA_RHA_MauBI_R | GGCGTC**CGCGCGCG**TTATCCAGTTATCAATTATAATAACTC | Reverse primer to clone in RHA into *pheA* knock-out vector |
| purD_LHA_MreI_F | GCTGGC**CGCCGGCG**AGATTTTACTCATAGGTTCA | Forward primer to clone in LHA into *purD* knock-out vector |
| purD_LHA_FspAI_R | GGCGTC**ATGCGCAT**AAGATACACACCCTTTTTAG | Reverse primer to clone in LHA into *purD* knock-out vector |
| purD_RHA_SgrDI_F | GCTGGCC**GTCGACG**TAAAGAAGAAAATATACATAAAATTAAAAAA | Forward primer to clone in RHA into *purD* knock-out vector |
| purD_RHA_MauBI_R | GGCGTCC**GCGCGCG**GTTTTAAAATTATTTTCTATAACGCC | Reverse primer to clone in RHA into *purD* knock-out vector |
| argH_cLHA_SbfI_F | GCTGGC**CCTGCAGG**AGTACAACTTATGATACAGAT | Forward primer to clone in LHA into *argH* complementation vector |
| argH_cLHA_NotI_R | GGCGTC**GCGGCCGC**TTACATTATTTTTTTATTTCCC | Reverse primer to clone in LHA into *argH* complementation vector |
| argH_cRHA_NheI_F | GCTGGC**GCTAGC**AGCATAGGGTTTTCTTAGG | Forward primer to clone in RHA into *argH* complementation vector |
| argH_cRHA_AscI_R | GGCGTC**GGCGCGCC**CAAAGTAGTCAATATTAGCAC | Reverse primer to clone in RHA into *argH* complementation vector |
| pheA_cLHA_SbfI_F | GCTGGC**CCTGCAGG**TAATCTTAATATAATTTATGAGC | Forward primer to clone in LHA into *pheA* complementation vector |
| pheA_cLHA_NotI_R | GGCGTC**GCGGCCGC**TTTATATTTTATAATAAGTACCTAC | Reverse primer to clone in LHA into *pheA* complementation vector |
| pheA_cRHA_NheI_F | GCTGGC**GCTAGC**AAATTATTTTGCCTTAAAGGAATAC | Forward primer to clone in RHA into *pheA* complementation vector |
| pheA_cRHA_AscI_R | GGCGTC**GGCGCGCC**CTCAATTGTAAAACCAAAGATA | Reverse primer to clone in RHA into *pheA* complementation vector |
| purD_cLHA_SbfI_F | GCTGGC**CCTGCAGG**AAAAAGGTTGTAATTGAAGAATATCTAG | Forward primer to clone in LHA into *purD* complementation vector |
| purD_cLHA_NotI_R | GGCGTC**GCGGCCGC**TTATTTAGATTTACCAATATCTTTTCTATAG | Reverse primer to clone in LHA into *purD* complementation vector |
| purD_cRHA_NheI_F | GCTGGC**GCTAGC**AGAAGAAAATATACATAAAATTAAAAAACT | Forward primer to clone in RHA into *purD* complementation vector |
| purD_cRHA_AscI_R | GGCGTC**GGCGCGCC**TCTTTAACACAAATTTCCTGTG | Reverse primer to clone in RHA into *purD* complementation vector |

**References (Supplementary Information)**

Cartman, S.T., Kelly, M.L., Heeg, D., Heap, J.T., Minton. N. P. 2012. Precise Manipulation of the *Clostridium difficile* Chromosome Reveals a Lack of Association between the *tcdC* Genotype and Toxin Production. *Appl Environ Microbiol.* 78**,** 13.

Ehsaan, M., Kuit, W., Zhang, Y., Cartman, S.T., Heap, J.T., Winzer, K., Minton. N. P. 2016. Mutant generation by allelic exchange and genome resequencing of the biobutanol organism *Clostridium acetobutylicum* ATCC 824. *Biotechnol Biofuels.* 9**,** 4.

Heap J.T., Pennington O.J., Cartman S.T., Minton N.P. 2009 A modular system for *Clostridium* shuttle plasmids, *J Microbiol Methods.* 78, 79-85

Ng, Y. K., Ehsaan, M., Philip, S., Collery, M. M., Janoir, C., Collignon, A., Cartman, S. T., & Minton, N. P. 2013. Expanding the repertoire of gene tools for precise manipulation of the *Clostridium difficile* genome: allelic exchange using *pyrE* alleles. *PloS One.* 8, e56051.

**
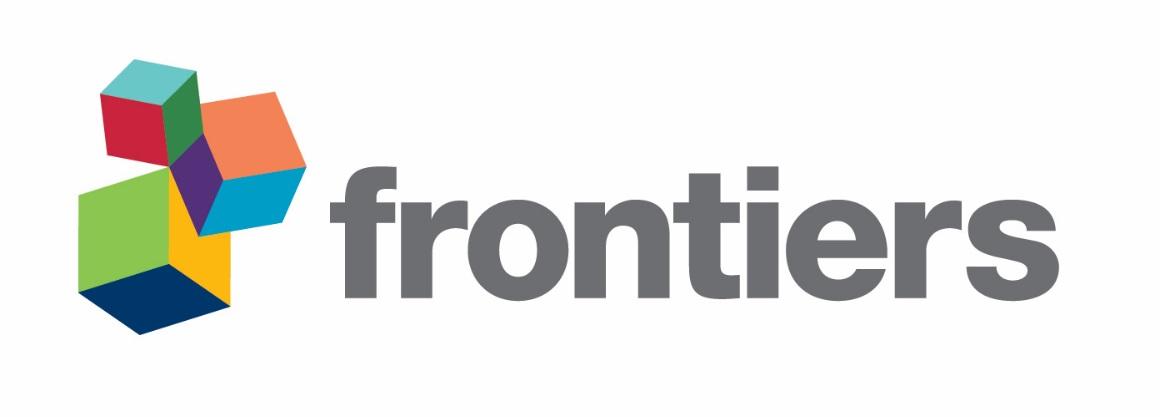
**
